# Open Brain AI and language assessment

**DOI:** 10.3389/fnhum.2024.1421435

**Published:** 2024-08-06

**Authors:** Charalambos Themistocleous

**Affiliations:** Department of Special Needs Education, Helga Engs Hus, University of Oslo, Oslo, Norway

**Keywords:** Open Brain AI, clinical AI analysis, language, cognition, natural language processing (NLP)

## Abstract

Neurolinguistic assessments play a vital role in neurological examinations, revealing a wide range of language and communication impairments associated with developmental disorders and acquired neurological conditions. Yet, a thorough neurolinguistic assessment is time-consuming and laborious and takes valuable resources from other tasks. To empower clinicians, healthcare providers, and researchers, we have developed Open Brain AI (OBAI). The aim of this computational platform is twofold. First, it aims to provide advanced AI tools to facilitate spoken and written language analysis, automate the analysis process, and reduce the workload associated with time-consuming tasks. The platform currently incorporates multilingual tools for English, Danish, Dutch, Finnish, French, German, Greek, Italian, Norwegian, Polish, Portuguese, Romanian, Russian, Spanish, and Swedish. The tools involve models for (i) audio transcription, (ii) automatic translation, (iii) grammar error correction, (iv) transcription to the International Phonetic Alphabet, (v) readability scoring, (vi) phonology, morphology, syntax, semantic measures (e.g., counts and proportions), and lexical measures. Second, it aims to support clinicians in conducting their research and automating everyday tasks with “OBAI Companion,” an AI language assistant that facilitates language processing, such as structuring, summarizing, and editing texts. OBAI also provides tools for automating spelling and phonology scoring. This paper reviews OBAI’s underlying architectures and applications and shows how OBAI can help professionals focus on higher-value activities, such as therapeutic interventions.

## 1 Introduction

Individuals presenting with neurological and behavioral challenges frequently remain undiagnosed due to insufficient specialized knowledge among family members and educators, particularly in the early stages of conditions such as Mild Cognitive Impairment (MCI). This lack of awareness and potential hesitancy to seek neurological evaluation can result in delayed assessment and intervention, potentially impacting long-term outcomes. Thus, there is a need to provide quick and accessible screening for neurological conditions affecting language. Additionally, supporting clinicians through automated analysis may enhance the understanding of the nuances of language disorders, facilitate treatment planning, and enable tracking progress throughout treatment.

Open Brain AI (OBAI)^1^ is a comprehensive computational platform that aims to support neurologists, clinicians, and researchers by providing flexible Artificial Intelligence (AI) language analysis solutions for spoken and written language. OBAI aims to facilitate clinicians and researchers in providing early screening processes and clinical feedback, offering significant advantages in neurocognitive research and clinical work. OBAI analyzes multimodal data, namely texts and sound recordings in several languages: English, Danish, Dutch, Finnish, French, German, Greek, Italian, Norwegian, Polish, Portuguese, Romanian, Russian, Spanish, and Swedish. Furthermore, the OBAI Companion, a large language model in OBAI, aims to ease the administrative burden on clinicians and researchers by providing writing assistance, such as feedback for improved clarity and precision, as well as generating draft documents and emails, saving valuable time and allowing clinicians to focus more on direct patient care.

In the following, we discuss how OBAI can be employed to analyze speech and language and identify language impairments associated with neurocognitive conditions. Specifically, we describe the modules for automatic transcription and translation, automatic scoring and quantification of speech productions, automatic assessment for language impairments (including grammatical error measurement), and language production measures. OBAI focuses on two primary user cases:

(1) Clinical Centers: These healthcare facilities support a broad spectrum of patients, including those with neurological, psychiatric, and developmental conditions, by providing a computational Application Programming Interface (API), which follows the safety and data protection standards. Clinical centers continually seek innovative, efficient tools to enhance their screening processes, improve diagnostic accuracy, and tailor interventions. OBAI API can be utilized in the development of a comprehensive ecosystem supporting a wide array of telehealth services:
  1. Teleconsultation: It can facilitate communication between healthcare professionals and remote patients.
  2. Telemonitoring: It can integrate with other health monitoring devices, offering a holistic view of a patient’s health by tracking changes over time, thus painting a comprehensive picture of their well-being.
  3. Teletherapy: It can bridge the gap between patients and essential therapy services, delivering speech-language pathology, audiology, and other therapeutic services directly to patients, wherever they are.
(2) Clinicians and Researchers: Given the growing awareness of cognitive health and millions of individuals seeking solutions to address their concerns, the OBAI web application provides access to tools and information to monitor cognitive status. OBAI supports clinicians and researchers with automated language analysis tools. It provides a user-friendly web interface that allows clinicians and researchers to quickly analyze data, gain insights, diagnose, predict prognosis, track disease progression, and measure treatment effectiveness. These tools can enable them to analyze substantial amounts of data quickly and efficiently and thus support diagnosis, prognosis, disease progression, and treatment efficacy.

OBAI relies on Natural Language Processing (NLP), Machine Learning (ML), Speech-to-Text transcription, and statistical and probabilistic models. The automatic methods allow for the reproducibility of the results and the standardization of measurements. Standardization promotes the precision of measures across studies, patients, and time points (Stark et al., 2020, 2022). For example, machine learning (ML) models like Random Forests and Neural Networks, along with tools such as morphosyntactic taggers and parsers, offer consistent and reproducible analysis of textual data when provided with the same code, data, and training process. This contrasts with human analysis, which can vary depending on the individuals and their time limitations.

The Open Brain AI Environment has four main modules. Module 1: the OBAI Text Editor; Module 2: the OBAI Companion; Module 3: the Language Analysis Options; and Module 4: Advanced Clinical Tools. Each module corresponds to an interface in the web application. The OBAI Text Editor in Module 1 is the primary interface. Two buttons on the top toolbar, “Companion” and “Options”, allow the user to access Module 2 and Module 3. Module 4 is accessible from Module 3: the Language Analysis Options.

## 2 Module 1: OBAI text editor

**FIGURE 1:**
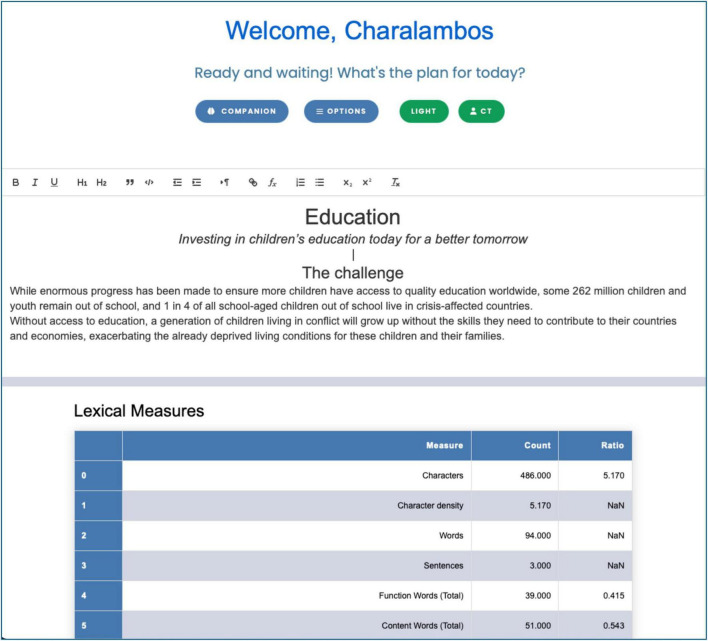
Main Interface of OBAI. The main OBAI working canvas is divided into a text editor (top) and a results area (bottom), separated by a draggable horizontal bar to adjust their relative heights. Users can interact with a text using the OBAI text editor. Results in the results area update dynamically as the text is edited. The text in the screenshot is from UNICEF’s website (https://www.unicef.org/eu/education, January 5, 2024).

The main OBAI working canvas (Figure 1) is divided into a text editor (top) and a results area (bottom), separated by a draggable horizontal bar to adjust their relative heights. The OBAI text editor is a central hub for users to interact with OBAI’s speech and language analysis features. The text editor facilitates creating, storing, and retrieving text documents. It also enables users to compose, analyze, and save texts. Moreover, it allows clinicians and researchers to utilize the platform’s capabilities for various research and analysis tasks. Users can type or copy a text in the OBAI Text Editor. Alternatively, they can use the Options to load a text from the computer or a text stored in OBAI (using the button with the user’s initials in the toolbar; CT in Figure 1 is the author’s initials). Users can export the text as a Microsoft Word document (docx) for further analysis using external text editors or save it in OBAI.

The Results Area under the horizontal dividing bar (Figure 1) serves as a hub for the output of the textual analysis from the Module 3: the Language Analysis Options or the output provided by Module 2: the OBAI Companion. Depending on the analysis performed (e.g., translation, grammar errors, and phonological analysis), the output can be presented in this space as a table or in a text box, which contains the more narrative-driven output. The text in textboxes is designed to be interactive. First, it allows the user to click on the box to enter the text back to the text editor for further analysis. This interactive functionality aims to improve the user experience, making working with the output text easier. Second, the results in output update dynamically as the text is edited in the text editor, providing information based on the options that are selected in Module 3.

## 3 Module 2: OBAI Companion

**FIGURE 2:**
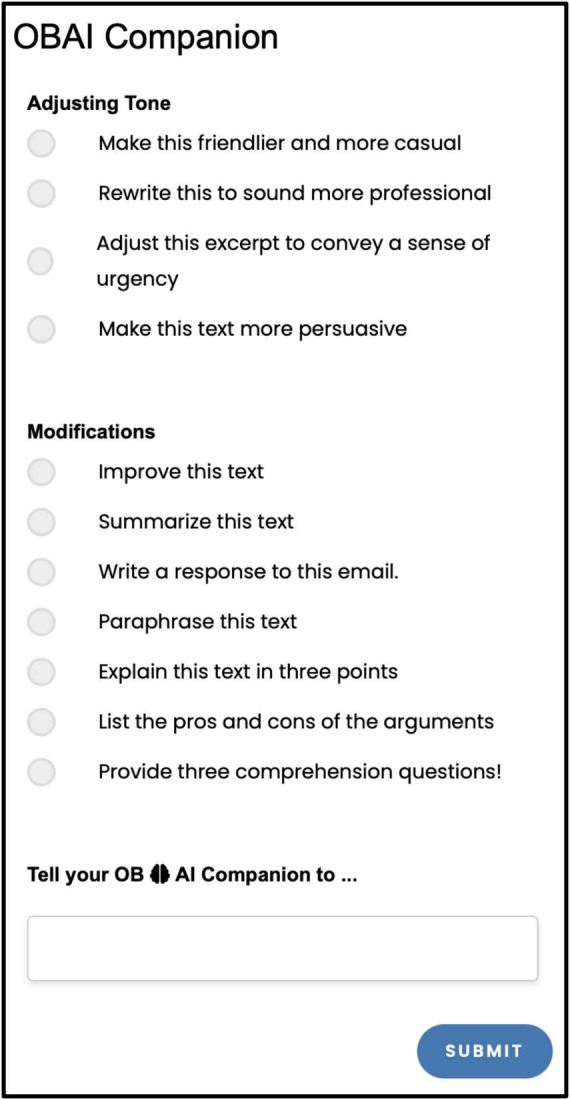
OBAI Companion; the interface in OBAI.

The Module 2: OBAI Companion (Figure 2) is an AI assistant that facilitates processing, understanding, and generating human language using a pre-trained language model. Clinicians and researchers can use the OBAI Companion as an open prompt for (1) examining patient transcripts or (2) evaluating grammar and style. Moreover, the OBAI Companion integrates with the text in the OBAI Text Editor and provides AI output to allow clinicians and researchers to perform clinical work, such as drafting reports, analyzing and improving the content and argumentation of their reports, checking the grammar and style mechanics in their texts, and getting feedback on their writing clarity and precision. The OBAI Companion offers two types of fixed prompts for adjusting the tone and modifying the text (Figure 2) and an open-ended prompt, a typing area, that users can use to interact with the OBAI Companion.

The OBAI Companion is a Large Language Model (LLM), which is currently based on Google Gemini. LLMs are constantly improving, following early proposals of language models, such as BERT (Vaswani et al., 2017; Devlin et al., 2018) and GPT2 (Radford et al., 2019). LLMs, like Google Gemini and ChatGPT, are designed to understand, generate, and interact with human language. Overall, LLMs have been widely recognized for their ability to perform a diverse range of tasks, including generating and summarizing texts like articles, poems, and email responses, preparing to-do lists, and enabling automatic translation.

## 4 Module 3: language analysis options

**FIGURE 3:**
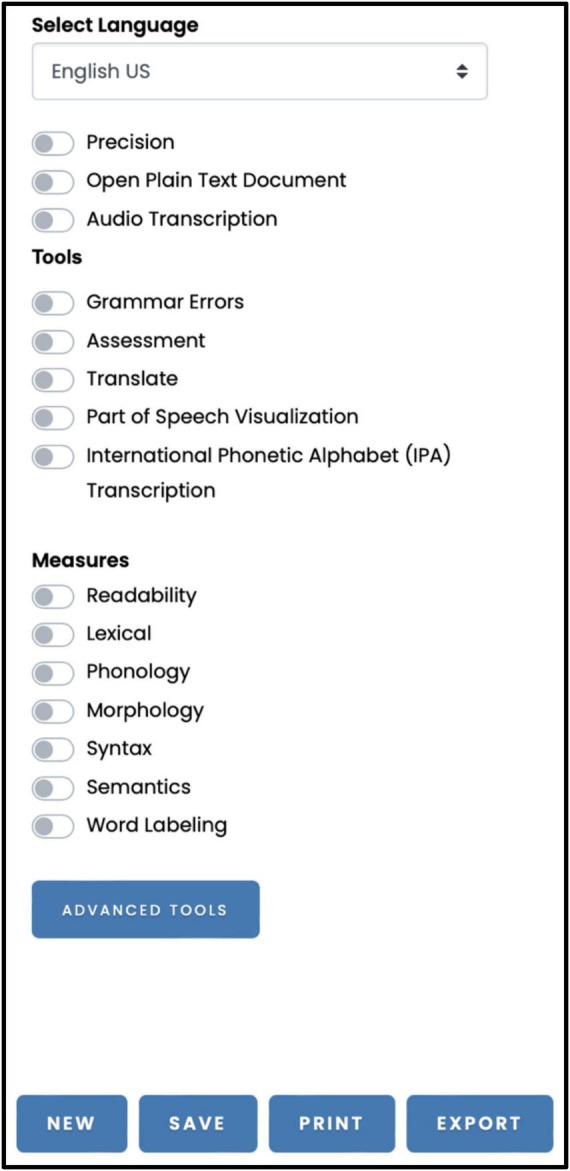
The Language Analysis Options interface offers a variety of features accessible from top to bottom: (i) Language Selection and Precision: Users can choose the language of their document or audio, and set the desired level of analysis precision; (ii) Input Options: Upload a document for text analysis or an audio file for transcription; (iii) Analysis Tools: Grammar & Spelling Check, Overall Text Assessment, Automatic Translation, Part-of-Speech Visualization, International Phonetic Alphabet Transcription, Linguistic Analysis (Readability, Lexical, Phonology, Morphology, Syntax, Semantics, Word Labeling); and (iv) Document Manipulation: Create, save, and export documents in various formats. The Advanced Tools button provides access to Module 4: Advanced Clinical Tools.

Module 3: Language Analysis Options allows the selection of language analysis options (Figure 3). In the following, we discuss the specifications of the Language Analysis Options module, discuss how to use OBAI for conducting linguistic analysis, and the available types of linguistic analysis that can be conducted through this Module.

### 4.1 Select language

Before analyzing a text, the user must select the corresponding language of the text from the dropdown menu in Module 3: Language Analysis Options. The subsequent analysis will be based on this language selection. For example, if the text in the editor is in Italian but the selected language is English, the analysis will use an English language model, resulting in incorrect output.

### 4.2 Precision

Precision will force OBAI to employ a more extensive and slightly more accurate NLP model when selected. This option affects the analysis of morphology, syntax, and semantics. OBAI currently employs open-source grammar models from the spacy package for the NLP analysis (Honnibal and Montani, 2017) and the Natural Language Toolkit (NLTK) (Hardeniya et al., 2016); the scores of the current models (June 2024) for all languages are provided in the Supplementary appendix. Usually, the [−] Precision results in faster processing and [++] Precision in a slightly more accurate model; see Table 1 that provides the model evaluation scores for English.

**TABLE 1:** NLP model for English without and with Precision score selected.

	[−] Precision	[+] Precision
Tokenization (Recall)	1	1
Tokenization (Precision)	1	1
Tokenization (F1)	1	1
Tokenization (Accuracy)	1	1
Sentence Boundary Detection (Recall)	0.89	0.86
Sentence Boundary Detection (Precision)	0.92	0.95
Sentence Boundary Detection (F1-Score)	0.91	0.9
POS Tagging Accuracy	0.97	0.98
Named Entity Recognition (Recall)	0.86	0.9
Named Entity Recognition (Precision)	0.85	0.9
Named Entity Recognition (F1-score)	0.85	0.9
Labeled Dependency Accuracy	0.9	0.94
Unlabeled Dependency Accuracy	0.92	0.95

The measurements in Table 1 correspond to the components or subsystems of the English NLP analysis (Honnibal and Montani, 2017). These components participate in analyzing the text, which involves processes such as finding the boundaries of words and sentences, labeling the part of speech of each word, and finding the syntactic constituents and semantic entities (e.g., person, organization, date). Tokenization is the process of breaking down sentences into words. Sentence Boundary Detection is identifying where sentences begin and end in a text. Part of Speech (POS) Tagging labels each word in a sentence with its part of speech (e.g., noun, verb, and adjective) (see also the Morphology section below). Named Entity Recognition identifies and classifies proper names in text (e.g., people, places, organizations) (see also the Semantics section below).

Dependency Parsing is the process of analyzing the grammatical structure of a sentence and establishing relationships between “head” words and words that modify those heads (see also Syntax section below). The Labeled Dependency Accuracy measures how often the model correctly identifies the grammatical relationships between words and labels those relationships correctly. High Accuracy means the model usually gets the relationships and labels right. Unlabeled Dependency Accuracy measures how often the model correctly identifies the relationships between words without considering the labels. High Accuracy means the model usually gets the relationships right, even if it sometimes mislabels them. The performance metrics provided in Table 1 include the precision (i.e., how many of the items the model finds are correct) and recall (how well the model finds all the correct items); the F1 Score balances recall and precision to give a single measure of performance; and the Accuracy informs about the correctness of the models.

### 4.3 Open plain text document

This option allows the user the user to open a plain text document (*.txt) and load it into the editor.

### 4.4 Audio transcription

OBAI speech-to-text aims to automate transcription of multilingual audio files. Users can upload a sound file and elicit an automated transcript, which can be passed to the editor for further analysis. The automatic transcription facilitates the process of creating clinical documentation by using transcribed sessions that can be quickly edited and organized into reports. Also, automatic transcription can be employed by clinicians and researchers as a feedback mechanism to help patients understand and visualize their speech patterns and errors in writing.

While highly effective, it is essential to acknowledge that automated transcription may face challenges with the speech of individuals affected by a speech and language disorder, such as developmental language disorder, dysarthria, stuttering, and apraxia of speech. In such cases, clinicians and researchers can still utilize the automatic transcription, but they should carefully review and adjust the output as needed. This supervised approach, particularly for speakers with significant speech impairments, often proves the most efficient in terms of time and resource allocation.

### 4.5 Grammar errors

The Grammar Errors provides an integrated grammar and style analysis, which includes information on grammatical errors in sentences, identifying complex sentence structures, run-on sentences, and fragments. It also provides information about potential phonemic paraphasias and neologisms (hapax legomena) by comparing each word to a dictionary. Clinicians and researchers can employ this module to analyze a text for errors or receive suggestions for improving a text for clarity and precision.

### 4.6 Assessment

The Assessment option employs AI language models to assess the text in the editor and tell whether there is evidence of it being produced by an individual with a speech and language impairment. It aims to provide a quick estimate or hint on whether a text deviates from healthy controls. The Assessment option lists briefly the criteria for making a certain estimate, namely if the text in the editor was authored by a person with a language impairment.

### 4.7 Translate

The translation component allows the translation of a text to twenty-one (21) languages (English (US), Bulgarian, Croatian, Czech, Danish, Dutch, Finnish, French, German, Greek, Hungarian, Italian, Norwegian Bokmäl, Polish, Portuguese, Romanian, Russian, Slovak, Spanish, Swedish, and Turkish). Crucial information may be inaccessible without practical translation tools, potentially compromising patient care. The Translate option aims to help clinicians and researchers overcome language barriers. Clinicians and researchers can translate research papers, case studies, patient records, and medical device instructions. Integrating translation directly into tools like OBAI offers access, saving valuable time in critical medical settings. As instructed by their organization (e.g., hospital or clinic), clinicians and researchers should always consult a professional translator in a medical setting.

### 4.8 Part of speech visualization

**FIGURE 4:**
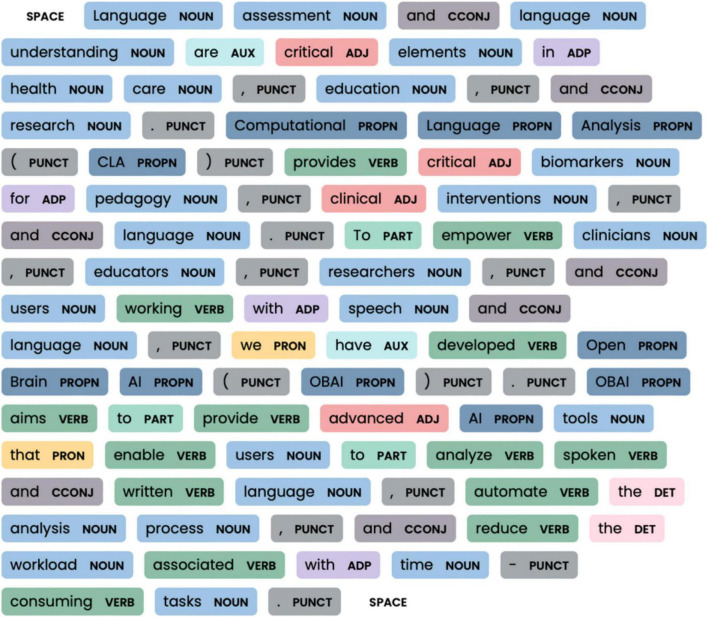
Morphological characterization of the text using colored labels.

By visualizing the grammatical analysis (Figure 4), Part-of-speech (POS) visualization assists in comprehending textual patterns, facilitates pattern identification, supports disambiguation, and aids in error detection. Part of speech visualization facilitates the comprehension of morphological structure, assisting clinicians and researchers in quickly identifying text’s grammatical structure and meaning. Also, it offers a quick picture to support the identification of patterns and relationships within text, such as the frequency of certain parts of speech or the distribution of nouns and verbs. In cases where words have multiple meanings, POS visualization can help disambiguate their intended usage and detect grammar errors. Lastly, clinicians and researchers can use the POS visualization figures in publications and educational and clinical materials to enhance the visual understanding of those texts.

### 4.9 International Phonetic Alphabet (IPA) transcription

The transcription to the International Phonetic Alphabet (IPA) allows users to convert a text in the OBAI Editor to phonemes. The IPA transcription is a grapheme to phoneme conversion; it does not simply convert a grapheme/letter to a phone but considers the phonological rules that apply at the lexical and post-lexical level; e.g., in the phrase, “is converted” /ɪz kənvˈɜːɾᵻd/ /s/ becomes /z/ in that phonemic environment due to lexical assimilation processes in English.

Text: “The input to the editor is converted to the IPA” (1)
IPA: /ðɪ ˈɪnpʊt tə ðɪ ˈɛDɪɾɚɹ ɪz kəNVˈɜːɾᵻd tə ðɪ ˌɪntɚnˈeɪʃənə fənˈɛɾɪk ˈælfəBˌɛt/

Grapheme-to-phoneme systems like this one generate a general phonemic representation, not a transcription specific to a particular speaker or recording. Nevertheless, by transcribing a text to IPA, clinicians and researchers can quantify the variations in sounds occurring within different phonemic contexts and identify the underlying patterns and structures of speech associated with speech pathology.

### 4.10 Measures

#### 4.10.1 Readability

Text readability measures are essential for quickly assessing written materials’ complexity (Klare, 1974). These measures can be beneficial in identifying text that may pose challenges for individuals with language impairment (Spadero, 1983). By using readability measures, clinicians and educators can tailor written materials to the appropriate reading level, ensuring they are accessible and comprehensible for their intended audience. For example, they can be employed in designing and administering tasks to individuals with reading comprehension difficulties (Senter and Smith, 1967). Also, clinicians can use readability measures to tailor their writing and make it more readable by their target audience with reading problems. Fitzsimmons et al. (2010) proposed the use of readability formulas to determine the accessibility of information provided by online consumer-orientated Parkinson’s disease (PD) websites as patients are employing the internet to access health information. The following commonly used readability measures exist in OBAI:

(I) Flesch reading ease

This measure calculates the ease of reading a text by considering the average sentence length and number of syllables per word. A higher score on this index indicates easier readability (Table 2). It can be used in education and content creation. For example, “the dog sleeps in the bedroom” would score high on the Flesch Reading Ease scale as it uses simple words and a short sentence length (Kincaid et al., 1975).

**TABLE 2:** Flesch Reading Ease Reading score levels and description.

Score	School level (US)	Description
100.00–90.00	5^th^ grade	Very easy to read
90.0–80.0	6^th^ grade	Easy to read
80.0–70.0	7^th^ grade	Fairly easy to read
70.0–60.0	8^th^ and 9^th^ grade	Standard Plain English
60.0–50.0	10^th^ to 12^th^ grade	Fairly difficult to read
50.0–30.0	College Level	Difficult to read
30.0–10.0	College Graduate	Difficult for the general population
10.0–0.0	Professional	Exceptionally difficult to read

Scores above one hundred (100) indicate simple texts, but we follow the standard convention and report 100 in this case.

$$206.835-1.015\times \frac{w\,o\,r\,d\,c\,o\,u\,n\,t}{s\,e\,n\,t\,e\,n\,c\,e\,c\,o\,u\,n\,t}-84.6\times \frac{s\,y\,l\,l\,a\,b\,l\,e\,c\,o\,u\,n\,t}{w\,o\,r\,d\,c\,o\,u\,n\,t}$$

(ii) Flesch-Kincaid grade level

This is a development of the Flesch Reading Ease measure that estimates the U.S. school grade level required to understand a text. It considers the average sentence length and number of syllables per word. For instance, “*the industrious professor assessed all student assignments diligently*” will likely correspond to a higher grade level due to more complex words and longer sentence length (Kincaid et al., 1975).

$$0.39\times \frac{w\,o\,r\,d\,c\,o\,u\,n\,t}{s\,e\,n\,t\,e\,n\,c\,e\,c\,o\,u\,n\,t}+11.8\times \frac{s\,y\,l\,l\,a\,b\,l\,e\,c\,o\,u\,n\,t}{w\,o\,r\,d\,c\,o\,u\,n\,t}-15.59$$

The Flesch-Kincaid Grade Level score is assessed on a scale. This is a standardized scale. A higher score indicates easier reading. A score of 5 means a fifth grader could understand the text easily. A score of 10 would require a high school sophomore reading level, whereas a score around eight is considered easy to read for most adults.

(iii) Gunning fog index

This index calculates readability by considering the average sentence length and the percentage of complex words (words with three or more syllables) in a text. A higher score on this index indicates a higher level of difficulty (Zhou et al., 2017). For example, “*the weather today is sunny.”* - This sentence would score low on the Gunning Fog Index as it uses simple words and has a short sentence length.

$$0.4\times \frac{w\,o\,r\,d\,c\,o\,u\,n\,t}{s\,e\,n\,t\,e\,n\,c\,e\,c\,o\,u\,n\,t}+100\times \frac{c\,o\,m\,p\,l\,e\,x\,w\,o\,r\,d\,s}{w\,o\,r\,d\,s}$$

(iv) Coleman-Liau index

The Coleman-Liau Index (Coleman and Liau, 1975) estimates the U.S. school grade level required to understand a text by considering the average number of characters per word and the average sentence length (Zhou et al., 2017). This is calculated as follows:

L = (letter count/word count) x 100
S = (sentence count/word count) x 100
Coleman-Liau Index is 0.0588 x L – 0.296 x S – 15.8

Thus, the Coleman-Liau Index relies on average sentence length and average number of characters per word (Table 3). The scale is like the Flesch scores: an index score of five (5) corresponds to a text produced by a fifth (5^th^) grader or below student, and it is very easy to read. Finally, an index score of six (6) corresponds to a sixth (6^th^) grader.

**TABLE 3:** Coleman-Liau Index levels and description.

Index Score	Grade	Difficulty
5	5^th^ grade and below	Very easy
6	6^th^ grade	Easy to read
7	7^th^ grade	Quite easy
10-Aug	8^th^ to 10^th^ grade	Conversational English
12-Nov	11^th^ and 12^th^ grade	Relatively hard
13– 16	College	Difficult
17+	Professional	Very hard

(v) Automated readability index

The Automated Readability Index estimates the U.S. school grade level required to understand a text by considering the average number of characters per word and the average sentence length (Senter and Smith, 1967; Kincaid et al., 1975). It is calculated as follows:

$$4.71\times \frac{c\,h\,a\,r\,a\,c\,t\,e\,r\,c\,o\,u\,n\,t}{w\,o\,r\,d\,c\,o\,u\,n\,t}+0.5\,\frac{w\,o\,r\,d\,c\,o\,u\,n\,t}{s\,e\,n\,t\,e\,n\,c\,e\,c\,o\,u\,n\,t}$$

A score of one (1) indicates 5- to 6-year-old children at the kindergarten level, a score of two (2) indicates 6- to 7-year-old children attending first grade, and a score of 14 indicates college students between 18 and 22, years old.

(vi) SMOG index

The SMOG (Simple Measure of Gobbledygook) Index estimates the years of education someone needs to understand a piece of writing easily (Fitzsimmons et al., 2010). It focuses on complex vocabulary. The index analyzes the number of words with three or more syllables in a text selection. First, the three ten-sentence-long samples from the text are selected, and words with three or more syllables (i.e., polysyllables words) are counted, and a grade is calculated with the following formula:

$$1.0430\,\sqrt{p\,o\,l\,y\,s\,y\,l\,l\,a\,b\,l\,e\,w\,o\,r\,d\,c\,o\,u\,n\,t\times \frac{30}{s\,e\,n\,t\,e\,n\,c\,e\,c\,o\,u\,n\,t}}+3.1291$$

A SMOG index score of eight (8) indicates that an individual would need an 8th-grade education level to comprehend the text.

(vii) Linsear write formula

The Linsear Write Formula focuses on sentence structure and word complexity (Eltorai et al., 2015). It is designed with shorter documents, specifically in mind. It uses a formula based on the number of sentences, words, and complex words (three or more syllables) found in a text sample. The standard Linsear Write metric *Lw* runs on a 100-word sample:

1. For each word with 2 syllables or less, add 1 point.
2. For a word with 3 syllables or more, add 3 points.
3. Divide the points by the number of sentences in the 100-word sample.
4. Adjust the provisional result *r*:

o If *r* > 20, *Lw* = *r* / 2.
o If *r* ≤ 20, *Lw* = *r* / 2 – 1.

Like the measures discussed above, the reported outcome corresponds to a “grade level” measure, reflecting the estimated years of education needed to read the text fluently. A higher Linsear Write score generally indicates easier readability. However, the raw score itself has less meaning than when compared with grade-level standards provided by Linsear Write.

(viii) Passive sentences percent

This metric calculates the percentage of sentences in a writing written in the passive voice. Although the degree of passive voice use is a contested issue, a high percentage of passive sentences can indicate a dense and less engaging text and, sometimes, a lower percentage can improve readability (Ownby, 2005). A 50% score indicates that half of the sentences are passive.

(ix) Dale Chall readability score

The Dale Chall score considers the revised wordlist of 3000 “easy words” that most fourth-grade students could understand (Chall and Dale, 1995). It measures the average sentence length and the percentage of words outside the “easy words” wordlist. The score corresponds to a U.S. grade-level reading requirement. For example, a score of 6.5 indicates the text should be understandable by someone at a 6th or 7th-grade reading level (Dale and Chall, 1948).

$$0.1579\,\left(\frac{d\,i\,f\,f\,i\,c\,u\,l\,t\,w\,o\,r\,d\,s\,c\,o\,u\,n\,t}{w\,o\,r\,d\,c\,o\,u\,n\,t}\times 100\right)+0.0496\,\left(\frac{w\,o\,r\,d\,c\,o\,u\,n\,t}{s\,e\,n\,t\,e\,n\,c\,e\,c\,o\,u\,n\,t}\right)$$

If the percentage of difficult words is above 5%, then 3.6365 is used to adjust the score, otherwise, the adjusted score is equal to the raw score. Difficult words are all the words in the given text, which are not included in the wordlist.

(x) Difficult words

The Difficult Words provides a count of difficult words by excluding those that can be considered easy, using a predefined list of words, which can be downloaded from Open Brain AI’s support page.^2^

#### 4.10.2 Lexical measures

The Lexical Measures provide lexical diversity metrics from a given text. Each measure provides unique insights into the text’s complexity, diversity, and linguistic features.

– Characters: The total number of characters in the text.
– Character Density: The number of characters divided by the total number of words in the text. The character density indicates how long words are in a text, indicating a preference towards longer or shorter words.
– Words: The total number of words in the text.
– Sentences: The total number of sentences in the text.
– Function Words (Total): The text’s count and proportion of function words. The function words include the following parts of speech: Adposition, Auxiliary, Coordinating conjunction, Determiner, Interjection, Particle, Pronoun, and Subordinating conjunction. Content and function words metrics were proposed by Themistocleous et al. (2020b) for the study of agrammatism and anomia.
– Content Words (Total): The text’s count and proportion of content words (the part of speech words that are not included the function words (Themistocleous et al., 2020b).
– Mean Sentence Length: The average number of words per sentence in the text.
– Propositional Idea Density: Propositional idea density (PID) is calculated as the number of unique propositions divided by the total number of words in a text. A proposition is defined in syntactic terms as a statement that expresses a complete thought, and it typically consists of a subject, a verb, and an object. For example, the sentence “The cat sat on the mat” contains two propositions: “The cat sat” and “The cat was on the mat.” To calculate the PID of a text, we first calculate all the unique propositions in the text, namely the nominal subject, direct object, adjectival and adverbial clauses, open complement, closed complement, and relative clause. Then, we estimate the ratio of propositions to sentences. For example, if a text contains one hundred (100) unique propositions and 1000 words, then the PID of the text would be 0.1. As PID measures the complexity of a text, a text with a high PID is more complex than a text with a low PID.
– Type-Token Ratio (TTR): The ratio of unique words (types) to the total number of words (tokens) in the text, multiplied by 100 to convert it to a percentage (Johnson, 1939; Tweedie and Baayen, 1998).

$$\frac{\mathrm{len}\,\left(\mathrm{unique}\,\mathrm{types}\right)}{\mathrm{total}\,\mathrm{tokens}}\times 100$$

This is a common measure employed in the study of discourse. OBAI provides several measures related to TTR (Themistocleous, 2023).

– Corrected Type-Token Ratio (CTTR): An adjusted TTR that accounts for text length. CTTR adjusts the traditional TTR for text length, making it more comparable across texts of different sizes (Templin, 1957; Carroll, 1964; Yang et al., 2022).

$$\text{CTTR}=\frac{\mathrm{unique}\,\mathrm{types}}{\sqrt{2\,\mathrm{total}\,\mathrm{tokens}}}$$

– Maas’s TTR (A2): A measure sensitive to text length and lexical richness (Mass, 1972).

$$\frac{\log\left(\log\left(\mathrm{len}\,\left(\mathrm{unique}\,\_\,\mathrm{types}\right)\right)\right)}{\log\left(\log\left(\text{total\_tokens}\right)\right)}$$

– Mean Segmental Type-Token Ratio (MSTTR): MSTTR divides the text into segments of equal size (e.g., 100 tokens) and calculates the TTR for each segment (Manschreck et al., 1981). MSTTR is the average of these TTR values across all segments. It provides a more stable measure of lexical diversity by mitigating the length effect on TTR.

MSTTR=∑(unique⁢types⁢in⁢segmenttotal⁢tokens⁢in⁢⁢  the⁢  segment)number⁢of⁢segments

– Herdan’s C is a logarithmic measure of lexical diversity that compares the logarithm of unique types to the logarithm of total tokens (Herdan, 1960; Tweedie and Baayen, 1998). This measure provides another perspective on lexical richness, considering the logarithmic relationship between unique and total words.

#### 4.10.3 Phonology

The phonology measures module provides counts of syllables and the syllable-to-word ratio. Similarly, it gives the counts and distributions of phonemes in the text. These are estimated from the transcription to the IPA application; see the Transcription to IPA Application for more details.

#### 4.10.4 Morphology

Recent developments in NLP, in combination with machine learning and speech analysis, can facilitate multilevel discourse analysis and provide fast, efficient, and reliable quantification of speech, language, and communication (Asgari et al., 2017; Themistocleous et al., 2018; Tóth et al., 2018; Calzà et al., 2021). Past research has employed morphosyntactic analysis to characterize impairments in speech and language, such as agrammatism and anomia (Fraser et al., 2014; Themistocleous et al., 2020b). Agrammatism is a language impairment where individuals omit function words, such as conjunctions, pronouns, articles, prepositions, and grammatical morphemes, such as -ing and -ed. It is linked to damage in the left inferior frontal gyrus caused by conditions such as stroke, neurodegeneration, tumor, and traumatic brain injury (Miceli et al., 1984; Goodglass, 1997). The morphological analysis informs clinicians about the ability of individuals to form words and select the grammatical information associated with word structure, such as tense, aspect, and case. The syntactic analysis provides information about syntactic processing. Overall, morphosyntactic measures can inform both cognitive and linguistic processes and pathology (Afthinos et al., 2022).

OBAI provides morphological scores about the distribution of parts of speech (POS), such as the number and ratio of Adjectives, Adpositions, Adverbs, Auxiliaries, Coordinating Conjunctions, Determiners, Interjections, and Nouns.

#### 4.10.4 Syntax

The Syntax option provides quantified syntactic measures, such as counts of syntactic constituents like sentences and phrases (e.g., noun phrases, verb phrases, and prepositional phrases). These are estimated from language-specific computational grammars. The measures in OBAI are elicited from a dependency parser, which finds syntactic relations using a dependency grammar (Jurafsky and Martin, 2009). The parser analyzes the grammatical structure of a sentence by identifying the relationships—dependencies—between words starting from the root of the sentence, typically the main verb.

#### 4.10.5 Semantics

The Semantics option calculates semantic measures from the input text. These measures quantify the distribution of semantic entities in the text, such as Organizations, Persons, Products, and Quantity. The semantic analysis determines entity characteristics (e.g., person, location, and company) (Bengio and Heigold, 2014; Pennington et al., 2014; Sutskever et al., 2014) and it is calculated using Name Entity Recognition (NER), a process of information extraction that shows how semantic relationships are presented linguistically (Jurafsky and Martin, 2009). For example, Napoleon [Person] was the king of France [Place].

To assess atypical semantic patterns that may indicate conditions like anomia, clinicians and researchers can utilize semantic measures to assess the production and distribution of entities (e.g., persons, organizations, locations) in patient speech or writing. By analyzing these patterns, clinicians and researchers can determine the patient’s cognitive and linguistic abilities, facilitating early detection and diagnosis of potential neurological issues. Moreover, regular semantic analysis of patient language can be instrumental in identifying personalized therapeutic targets and tracking progress over time.

#### 4.10.6 Word labeling

The word labeling module enhances clinical research by comprehensively characterizing each word in each text (Table 4). The output is a table with the words of the text in the first column; the second column provides the syllabification. Namely, it shows the breaking down of the word into syllables. The third column provides the lemma, which is the base form of the word (e.g., “invest” for “investing”). Next to the lemma is the POS column, which shows the POS category, such as nouns, verbs, and adjectives.

**TABLE 4:** Example of the word-labeling output for the sentence “My friend’s house is always decorated beautifully.”

Word	Syllables	Lemma	POS	POS Details	Dependencies
My	‘my’	my	Pronoun	Pronoun, Possessive	Possession Modifier
Friend	‘friend’	friend	Noun	Noun, Singular or Mass	Possession Modifier
‘S	None	‘s	Particle	Possessive Ending	Case Marking
House	‘house’	house	Noun	Noun, Singular or Mass	Nominal Subject (Passive)
Is	‘is’	be	Auxiliary	Verb, 3Rd Person Singular Present	Auxiliary (Passive)
Always	‘al’, ‘ways’	always	Adverb	Adverb	Adverbial Modifier
Decorated	‘dec’, ‘o’, ‘rat’, ‘ed’	decorate	Verb	Verb, Past Participle	Root
Beautifully	‘beau’, ‘ti’, ‘ful’, ‘ly’	beautifully	Adverb	Adverb	Adverbial Modifier

Finally, it provides the Syntactic Dependencies: Analyzing the relationships between words in a sentence (e.g., subject-verb, noun-adjective) provides insights into how individuals construct meaning and express thoughts, revealing potential disruptions in language processing due to neurological conditions. Word Labeling allows users to evaluate the scores provided in those sections.

## 5 Module 4: Advanced Clinical Tools

**FIGURE 5:**
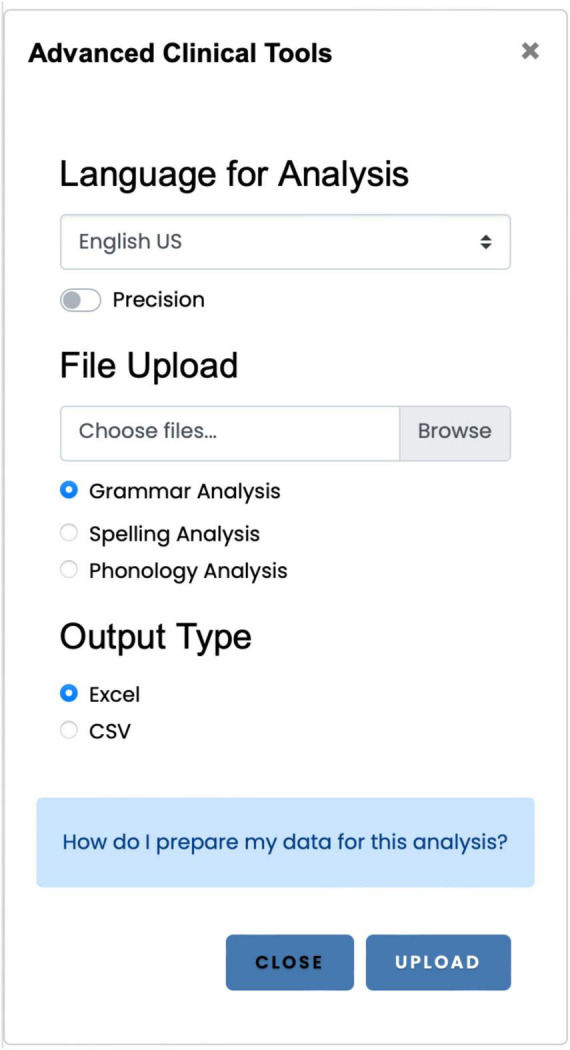
The Advanced Clinical Tools is the primary interface for accessing the three primary applications: grammar analysis, spelling, and the phonology scoring applications.

The Advanced Clinical Tools (Figure 5) provides access to three applications: the grammar analysis application, the automatic spelling analysis, and the phonology analysis application. The Advanced Clinical Tools allow users to process one or more spreadsheets (spelling and phonology application) or texts (grammar analysis) and export the measures in a spreadsheet.

### 5.1 Grammar analysis

Using the Grammar Analysis option, users can upload one or more plain text documents for linguistic analysis. The application will perform morphosyntactic processing, extracting each document’s phonological, morphological, syntactic, and semantic measures. These results are then presented in a structured table format (e.g., Excel or CSV) for review and further quantitative analysis.

### 5.2 Spelling analysis (Spelling scoring application)

The evaluation of spelling is a complex, challenging, and time-consuming process. To determine spelling errors, we have developed a spelling distance algorithm that quantifies spelling errors automatically. It relies on comparing letter-to-letter, the words spelled by the patients to the target words, using the Levenshtein Distance (Hixon et al., 2011). It processes both words and non-words (Themistocleous et al., 2020a). The algorithm automatically compares the inversions, insertions, deletions, and transpositions required to make the target word and response identical (Themistocleous et al., 2020a). It has two modes, namely, it scores words and non-words, i.e., pseudoword constructions. Words are not changed before comparison, whereas non-words are converted first to phonemes following the IPA and then compared. This approach accounts for the fact that non-words do not have standardized orthography (Caramazza and Miceli, 1990; Tainturier and Rapp, 2001, 2003). Thus, writers rely on their phonemic buffer to convert the words rather than on what they know from applying conventional orthography.

In addition to providing a composite score, like the work by Themistocleous et al. (2020a), the OBAI spelling application offers a detailed analysis of spelling errors, including inversions, insertions, deletions, and transpositions, on a word-by-word basis. This matches manual clinical scoring methods that also provide scores for errors, such as insertions and deletions (Caramazza and Miceli, 1990; Tainturier and Rapp, 2001, 2003). Finally, OBAI spelling analysis application is multilingual support for English US, Danish, Dutch, Finnish, French, German, Greek, Italian, Norwegian, Polish, Portuguese, Romanian, Russian, Spanish, and Swedish.

### 5.3 Phonological analysis (phonology scoring application)

The Phonological Analysis application converts the target and response words into phonemes using the International Phonetic Alphabet and compares their differences using the Levenshtein Distance (Hixon et al., 2011). Finally, it provides the composite phonemic score and scores of phonemic errors, namely deletions, insertions, transpositions, and substitutions. Lastly, it offers multilingual support, supporting the same languages as the Spelling Analysis application. The Phonological Analysis can be an essential clinical tool, especially for clinicians who manually transcribe and score phonological errors, offering quick and objective phonological scores.

## 6 Applications of Open Brain AI

In the previous section, we detailed the linguistic measures provided by OBAI. These measures have the potential to aid in the diagnosis, prognosis, and evaluation of therapy for language disorders. In this section, we illustrate the applications of these measures with examples from patients with neurodegenerative disorders that affect language.

Automated measures from speech and language provided by OBAI can be employed for diagnosis and prognosis. For example, Themistocleous et al. (2021) employed morphosyntactic measures, which were elicited from a picture description task to subtype individuals with Primary Progressive Aphasia (PPA), a neurodegenerative condition that affects speech and language into three variants: non-fluent variant PPA (nfvPPA), semantic variant PPA (svPPA), and logopenic PPA (lvPPA) (Gorno-Tempini et al., 2011). Subsequently, Themistocleous et al. (2021) trained a feedforward neural network, which was able to classify patients with PPA into variants with 80% classification accuracy. In an earlier study, Fraser et al. (2014) employed natural language processing to elicit textual measures (e.g., number of words, morphosyntactic and syntactic complexity measures). Fraser et al. (2014) demonstrated that morphosyntactic measures can distinguish individuals with semantic dementia (SD), progressive nonfluent aphasia (PNFA), and healthy controls.

OBAI lexical, morphosyntactic, and textual features from connected speech productions provided language biomarkers associated with patients with PPA. For example, Themistocleous et al. (2020b) analyzed connected speech productions from 52 individuals with PPA using a morphological tagger, an NLP algorithm implemented in OBAI, which automatically provides the part of speech category label for all words that individuals produce (Bird et al., 2009). From the tagged corpus, they measured both content words (e.g., nouns, verbs, adjectives, adverbs) and function words (conjunctions, e.g., *and*, *or*, and *but*; prepositions, e.g., *in*, and *of*; determiners, e.g., *the, a/an, both*; pronouns, e.g., *he/she/it* and wh-pronouns, e.g., *what, who, whom*; modal verbs, e.g., *can, should, will*; possessive endings (*‘s*), adverbial particles, e.g., *about, off, up*, and infinitival *to*, as in *to do*). Themistocleous et al. (2020b) showed that the POS patterns of individuals with PPA were both expected and unexpected. It showed that individuals with nfvPPA produced more content words than function words. Individuals with nfvPPA produced fewer grammatical words than those with lvPPA and svPPA, a critical symptom of agrammatism that characterizes these patients.

Additionally, OBAI’s automated NLP functionality can facilitate large-scale discourse analysis, providing an efficient and scalable approach for identifying linguistic patterns and markers associated with various communication disorders. A significant advantage of discourse and conversation analysis lies in their ecological validity, providing a more naturalistic assessment of language function than contrived, closed-set linguistic tasks. This approach captures the dynamic interplay of language skills within authentic communicative contexts, offering valuable insights into the manifestations of language symptoms. Longitudinal studies examining the discourse micro- and macro-structures within autobiographies of the School Sisters of Notre Dame congregation (Danner et al., 2001) revealed deteriorating patterns in linguistic expression in nuns of the congregation who developed dementia later in their lives. Furthermore, comparative discourse analyses of prominent figures such as British novelists Iris Murdoch and Agatha Christie (Le et al., 2011), as well as U.S. President Ronald Reagan (Berisha et al., 2014, 2015), have indicated that quantifiable metrics of lexico-syntactic complexity and idea density may serve as early markers of cognitive decline.

## 7 Discussion

Neurolinguistic assessment involves a variety of tasks such as naming (identifying objects from verbal or visual cues), fluency (spontaneously generating words or sentences), grammar (understanding and using grammatical rules), and receptive language (comprehending spoken and written language) (Lezak, 1995). These tasks are integral to a comprehensive neurological assessment, which may also include MR imaging and assessment of visual perception, visuomotor skills and coordination, and executive functioning (planning, organizing, decision-making, and working memory), as well as assessments of learning, memory (immediate, short-term, and long-term), and attention (Lezak, 1995).

An advantage of OBAI is that it is primarily a clinical and research tool that integrates with existing neurocognitive assessments and has the potential to support diagnosis and provide biomarkers and scores in cross-sectional and longitudinal studies, including monitoring a patient’s language functioning over time and assessing treatment efficacy. Moreover, OBAI allows the analysis of complex texts, such as spoken and written discourse. Unlike standardized texts, discourse provides ecological measure of language, which correspond to everyday use of language. Thus, the analysis of discourse provides richer information about language communication and conversation that is not typically captured in standardized assessments (Garrard et al., 2014; Stark et al., 2020, 2022; Themistocleous, 2023). OBAI is continuously updated to include measures such as Propositional Idea Density, a standardized measure of the number of ideas expressed in the number of words or sentences (Danner et al., 2001; section Lexical measures). For example, Farias et al. (2012) employed idea density, a computational measure, to measure cognitive decline in the Nun Study, a longitudinal study of cognitive decline.

### 7.1 Ethics

OBAI aims to comply with data safety regulations in the United States and the European Union, particularly the Health Insurance Privacy and Accountability Act (HIPAA) and the General Data Protection Regulation (GDPR). Therefore, we minimize data collection, use temporary storage, and are transparent in our privacy policy for fair and lawful data handling. Currently, the only user-related information that OBAI saves is the registration information and the contents of the OBAI Text-Editor if the users choose to save them. Minimizing data collection and deleting it after processing adheres to Data Minimization and Storage Limitation principles by not retaining data longer than necessary from the analysis. Users have control over their data as we do not store transcribed text or other post-analysis information.

OBAI does not collect user data for training models or further analysis. Both data and the reports created by OBAI are removed from OBAI, including sounds for transcription, texts for elicitation of automatic grammar measures, and tables for spelling and phonology scoring. Text retained on our servers are only the primary working documents of users in the text editor if users decide to save them and access them at another session.

### 7.2 Limitations and future research

While integrating multiple subsystems effectively, OBAI presents two primary limitations. First, its resource-intensive nature can lead to memory constraints when analyzing lengthy or numerous texts, potentially returning a server error. This challenge can be mitigated by allocating additional resources. Secondly, while OBAI is designed to analyze speech and language from healthy individuals and patients, individual pathology characteristics can challenge the models discussed earlier. Also, factors such as premorbid literacy, multilingualism, and co-occurring diagnoses can significantly influence the results and must be carefully considered. Therefore, clinicians and researchers should always remain responsible for reviewing and supervising the output of AI-based assessment tools. Despite this, OBAI can significantly reduce the workload of clinicians and researchers by automating time-consuming tasks and providing valuable insights. OBAI is not a static model but improves over time capturing a wider range of language phenomena and language varieties (Themistocleous, 2016, 2019).

OBAI, originally developed and maintained by a single individual, has reached a point where further advancement necessitates a dedicated research group. This expansion will enable a broader development scope and ensure adequate supervision to cater to a global audience. We will focus on establishing a research group centered around OBAI, aiming to scale up and refine the platform’s computational language assessment tools. This collaborative approach will foster innovation, enhance functionality, and ultimately extend the reach and impact of OBAI within the field of Computational Neurolinguistic Assessment.

## Data availability statement

The original contributions presented in this study are included in this article/Supplementary material, further inquiries can be directed to the corresponding author.

## Author contributions

CT: Conceptualization, Data curation, Formal analysis, Funding acquisition, Investigation, Methodology, Project administration, Resources, Software, Supervision, Validation, Visualization, Writing–original draft, Writing–review and editing.

## Footnotes

1
http://openbrainai.com

2
https://openbrainai.com/support/

## Supplementary material

Supplementary Appendix Parser evaluation (2024).
